# Novel Domain Knowledge-Encoding Algorithm Enables Label-Efficient Deep Learning for Cardiac CT Segmentation to Guide Atrial Fibrillation Treatment in a Pilot Dataset

**DOI:** 10.3390/diagnostics14141538

**Published:** 2024-07-17

**Authors:** Prasanth Ganesan, Ruibin Feng, Brototo Deb, Fleur V. Y. Tjong, Albert J. Rogers, Samuel Ruipérez-Campillo, Sulaiman Somani, Paul Clopton, Tina Baykaner, Miguel Rodrigo, James Zou, Francois Haddad, Matei Zaharia, Sanjiv M. Narayan

**Affiliations:** 1 Department of Medicine and Stanford Cardiovascular Institute (CVI), Stanford University, Stanford, CA 94305, USA; prash030@stanford.edu (P.G.); ruibin@stanford.edu (R.F.);; 2 Heart Center, Department of Clinical and Experimental Cardiology, Amsterdam UMC, University of Amsterdam, 1105 AZ Amsterdam, The Netherlands; 3 Department of Computer Science, ETH Zurich, 8092 Zurich, Switzerland; 4 CoMMLab, Universitat de València, 46100 Valencia, Spain; 5 Department of Biomedical Data Science, Stanford University, Stanford, CA 94305, USA; 6 Department of Computer Science, University of California Berkeley, Berkeley, CA 94720, USA

**Keywords:** cardiac CT segmentation, machine learning, domain knowledge encoding, atrial fibrillation, ablation

## Abstract

**Background:** Segmenting computed tomography (CT) is crucial in various clinical applications, such as tailoring personalized cardiac ablation for managing cardiac arrhythmias. Automating segmentation through machine learning (ML) is hindered by the necessity for large, labeled training data, which can be challenging to obtain. This article proposes a novel approach for automated, robust labeling using domain knowledge to achieve high-performance segmentation by ML from a small training set. The approach, the domain knowledge-encoding (DOKEN) algorithm, reduces the reliance on large training datasets by encoding cardiac geometry while automatically labeling the training set. The method was validated in a hold-out dataset of CT results from an atrial fibrillation (AF) ablation study. **Methods:** The DOKEN algorithm parses left atrial (LA) structures, extracts “anatomical knowledge” by leveraging digital LA models (available publicly), and then applies this knowledge to achieve high ML segmentation performance with a small number of training samples. The DOKEN-labeled training set was used to train a nnU-Net deep neural network (DNN) model for segmenting cardiac CT in *N* = 20 patients. Subsequently, the method was tested in a hold-out set with *N* = 100 patients (five times larger than training set) who underwent AF ablation. **Results:** The DOKEN algorithm integrated with the nn-Unet model achieved high segmentation performance with few training samples, with a training to test ratio of 1:5. The Dice score of the DOKEN-enhanced model was 96.7% (IQR: 95.3% to 97.7%), with a median error in surface distance of boundaries of 1.51 mm (IQR: 0.72 to 3.12) and a mean centroid–boundary distance of 1.16 mm (95% CI: −4.57 to 6.89), similar to expert results (r = 0.99; *p* < 0.001). In digital hearts, the novel DOKEN approach segmented the LA structures with a mean difference for the centroid–boundary distances of −0.27 mm (95% CI: −3.87 to 3.33; r = 0.99; *p* < 0.0001). **Conclusions:** The proposed novel domain knowledge-encoding algorithm was able to perform the segmentation of six substructures of the LA, reducing the need for large training data sets. The combination of domain knowledge encoding and a machine learning approach could reduce the dependence of ML on large training datasets and could potentially be applied to AF ablation procedures and extended in the future to other imaging, 3D printing, and data science applications.

## 1. Introduction

The segmentation of cardiac computed tomography (CT) images has historically been performed by semi-automated algorithms such as graph-cuts [1], region growing [2] with manual seed inputs, and other traditional image-processing methods. Deep neural networks (DNN) showed superior performance to traditional image processing even for complex tasks such as segmenting a person of interest in an image of a crowded street [3] or classifying complex diseases from radiology scans [4,5]. However, DNN models require a large amount of training data, which, in the context of cardiac CT segmentation, is challenging to obtain. Several publicly available medical datasets include <100 cases [6,7,8] due to technical, privacy, and regulatory concerns. Since deep learning typically reserves the majority of cases for training, models are thus often tested on <40 cases [8], which may limit generalizability [9,10].

This begs the question as to whether high DNN performance can be obtained when the number of training samples is smaller than that of test-set samples. The focus of this work is to explore the idea of achieving high DNN segmentation performance in cardiac CT images from a small number of training samples. A DNN was applied to raw CT images to segment the left atrium (LA) body and other LA substructures: four pulmonary veins (PVs) and one LA appendage (LAA), which are central to treating patients with atrial fibrillation (AF). Although this paper focuses on a label-efficient segmentation approach of the six LA substructures for AF application, the approach can theoretically be extended to segment other chambers of the heart as well.

This article proposes a novel approach called the domain knowledge-encoding (DOKEN) algorithm, which extracts “anatomical knowledge” by leveraging digital LA models (available publicly) and then applies this knowledge to achieve high DNN segmentation performance with small number of training samples. The DOKEN algorithm essentially pre-processes the training samples before inputting them for DNN training. The pre-processing involves automatic labeling to obtain robust ground-truth labels of LA substructures. The performance of the DOKEN-labeled DNN model was tested in a hold-out dataset >5 times larger than the training set.

The purpose of this study is to test the hypothesis that ML models could be trained using very small datasets if combined with some domain knowledge of the task at hand. This method of training using conceptual domain knowledge principles rather than massive training data [11,12] is analogous to how humans can learn from small data [12]. Lake et al. used this approach to generate handwritten characters with human-level performance from one exemplar by parsing characters into simple primitives that were composited to create new characters [13]. However, for medical image analysis, such domain knowledge has rarely been used to reduce training sizes for DNN [14,15]. In the following sections, we describe the methods, results, discussion and conclusions.

## 2. Methods

**Figure 1:**
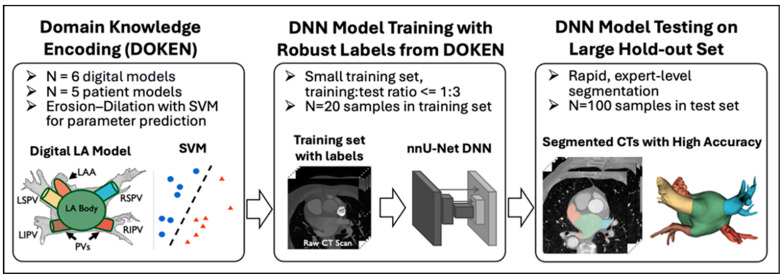
Method overview: proposed domain knowledge-encoding algorithm used to label CT images for efficient DNN training on a training set significantly smaller than the test set. LA: left atrium, LSPV: left superior pulmonary vein, LIPV: left inferior pulmonary vein, RSPV: right superior pulmonary. Each color represents an LA sub-structure.

Figure 1 outlines the method. (1) The proposed DOKEN algorithm encoded domain knowledge of the LA body and other anatomies; (2) the algorithm was used to train a nnU-Net DNN to segment cardiac CT images using only a small training set; and (3) the trained DNN was tested in a large hold-out set.

### 2.1. Dataset for Training and Testing

The CT dataset used in this study consists of *N* = 120 patients who had undergone AF ablation between October 2014 and July 2019 and had cardiac CT scans. All patients signed informed consent at Stanford Health Care. We split this dataset randomly into *N* = 20 for DNN model training (Training Set), with *N* = 100 patients as a hold-out test set (Test Set). Note that the number of samples in the training set is 5 times smaller than the test set samples, which is one of the key contributions of this study. Separately, for developing the DOKEN algorithm, *N* = 6 publicly available 3D digital heart models built using Gaussian process morphable models [16] was used.

### 2.2. Domain Knowledge-Encoding (DOKEN) Algorithm

**Figure 2:**
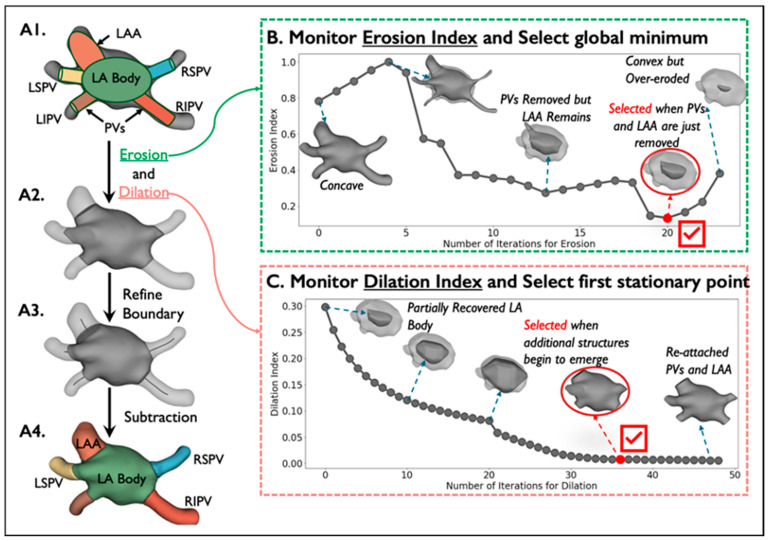
Segmentation of digital LA models by erosion–dilation: Detailed description of the first step of the DOKEN algorithm. (**A1**–**A4**) The pipeline of our DOKEN algorithm. (**B**,**C**) Iterative variations of the erosion and dilation indices along with variations in LA model corresponding to iterations. The ED parameters from this step are then learned by an SVM for labeling clinical data.

The goal of the DOKEN algorithm is to automatically generate robust ground-truth labels of LA substructures for DNN training. The algorithm consists of the following two steps:

I. Segmentation of digital LA models: *N* = 6 digital LA models (publicly available) were segmented based on an iterative erosion–dilation (ED) process (Figure 2).
II. Tuning ED parameter using patient LA models: The iterative ED process requires optimal iteration number as a parameter, which decides the accurate segmentation of the LA body and other substructures. To determine this parameter, 5 manually segmented LA models were used to train support vector machines (SVMs) to predict the optimal iteration in the ED process.

The two steps are used to develop the DOKEN algorithm, and once its developed, it takes training images as input and generates ground-truth labels as output. The two development steps are detailed below.

I. Segmentation of digital LA models

We reasoned that heart structures can be geometrically parsed by separating the convex LA body from the concave whole heart. Three-dimensional voxel erosion, dilation [17], and subtraction were used for this purpose.

To segment PV and LAA from the digital heart, a binary erosion operation was used, which can be defined as $A⊖B=\;\{x\in \;E^{N}\;|\;x+b\in A\;for\;every\;b\in B\}$. Then, in order to recover the original dimension of LA, binary dilation was applied, defined as $A⊕B=\;\{x\in \;E^{N}\;|\;c=a+b\;for\;some\;a\in \;A\;and\;b\in B\}$, where A and B are sets in N-space ($E^{N}$) with elements a and b. In our case, A is the heart model and B is a structuring element, which is a 3×3×3 cube where the center and its 6 neighbors are set to 1 and the remaining elements are 0s.

First, the digital shells were segmented by the application of erosion to concave junctions between PVs and LAA with the LA (Figure 2(A1)). The PVs and LAA are smaller and consist of more 1-connected voxels than the LA body and thus erode more rapidly. However, it is non-trivial to iteratively erode just the PVs and LAA to leave the residual convex LA. To do so, an *Erosion Index* was proposed to monitor the progression of erosion:

$$Erosion\;Index=\frac{V\left(Convex\left(E_{i}\right)\right)-V\left(E_{i}\right)}{V\left(E_{i}\right)},$$

where $E_{i}$ is the 3D model after the ith erosion, $Convex\left(\cdot \right)$ is the convex hull, and $V\left(\cdot \right)$ returns the volume of a 3D shape. The erosion index approaches 0 as the shape becomes convex. The index data are preprocessed with a Savitzky–Golay filter and fitted with a polynomial function. The global minimum of the fitting function is calculated to determine the number of iterations for erosion (Figure 2B).

Because erosion may remove outer layers of the LA, a dilation operation was applied to recover its original dimension (Figure 2(A2)) by paving voxels on the contour and stopping just before the PVs and LAA are re-attached (Figure 2(A3)), which is monitored by the proposed *Dilation Index* by measuring the number of added voxels after each dilation:

$$Dilation\;Index=\frac{V\left(D_{i+1}\right)-V\left(D_{i}\right)}{V\left(D_{i}\right)},$$

where $D_{i}$ is the 3D shell after the ith dilation and $V\left(\cdot \right)$ returns the volume of a 3D shape. Similarly, we processed the index data using a Savitzky–Golay filter then fitted them with a polynomial function. The first stationary point of the fitting function determines the number of dilation iterations (Figure 2C).

After the left atrium body is isolated after erosion and dilation, the boundaries between the LA body and the PVs and LAA were refined by calculating centerlines from the LA centroid to the centroid of each segmented structure. This approach has been used to extract and segment the aorta and great vessels [6,18,19]. Below is a step-by-step algorithm of boundary refinement and centerline calculation:

1. Extrapolate a Voronoi diagram [20] from the shell (Figure 2(A1)) to all internal points to create a maximal sphere centered at that point.
2. Calculate the centroid of the LA body and the centroid of each virtually dissected substructure (4 PVs and LAA).
3. For each substructure centroid, create a centerline automatically by minimizing the integral of the radius of maximal inscribed spheres along the path that connects the substructure centroid to the LA body centroid.
4. Replace the boundary between the left atrium and each substructure by a plane orthogonal to the corresponding centerline and close to the original boundary generated by the ED process (Figure 2(A4)).

II. Tuning the ED parameter using patient LA models

The parameters for the ED process, i.e., the optimal number of iterations, that are suitable for digital models may not apply to clinical data due to heterogeneities such as anatomy variability and imaging artifacts present in the clinical data. The parameters were made suitable for clinical data using a support vector machine (SVM) to predict the parameter value for input clinical CT. Two SVMs (one for each parameter) were trained with manually annotated seed samples (*N* = 5) to predict the optimal number of erosion and dilation iterations. The ED process with parameters predicted by SVMs forms the DOKEN algorithm and will be used to generate robust labels for training the DNN model.

### 2.3. Training the DNN for CT Segmentation from a Small Training Set

DOKEN was applied to *N* = 20 training data to label the different LA structures in each sample. This was used as ground truth for training the DNN.

**Figure 3:**
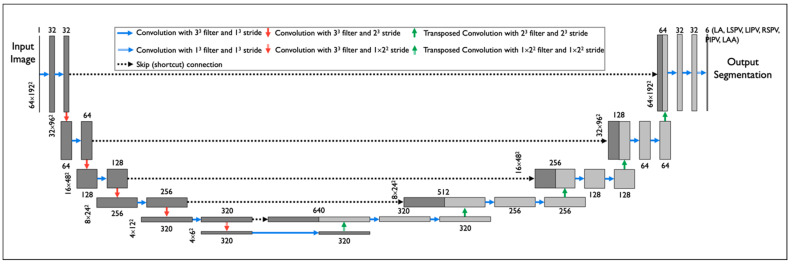
DNN model architecture: nn-Unet was applied to segment raw cardiac CT images. The model was trained using a DOKEN-labeled training set (small size) and was tested on a large hold-out test set.

**Figure 4:**
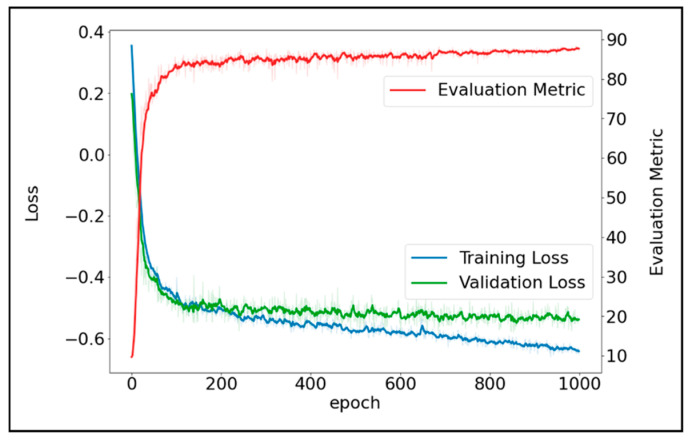
Convergence of training loss, validation loss, and the Dice validation accuracy.

We implemented nnU-Net (Figure 3)—a DNN model which has been widely used in 23 public datasets [21]. To train the nnU-Net model, first, each input CT scan was z-score normalized by subtracting its mean, followed by division by its standard deviation. Then the images were re-sampled using third-order spline interpolation. The target voxel spacing was set as the median spacing of the training samples. To improve the generalizability, a set of data augmentation techniques were randomly applied on the fly during training, including rotations, flipping, scaling, Gaussian noise and blur, and random changes in brightness, contrast, and gamma. During the training process, the batch size was set to 2 due to the GPU memory limitation, and the DL model was trained for 1000 epochs. Stochastic gradient descent [22] was used to optimize the model. The initial learning rate and Nesterov momentum were set to 0.01 and 0.99, respectively. The sum of cross-entropy and Dice loss were used as training loss. Figure 4 shows the convergence of training loss, validation loss, and validation accuracy (measured by Dice) during training.

### 2.4. Experimental Setting for Performance Evaluation

The DOKEN algorithm’s ability was empirically evaluated to parse cardiac geometry and the DNN model’s ability to segment cardiac structures from CT images. The large test set (*N* = 100) was used to manually annotate the ground-truth labels for the 6 substructures by a panel of clinical experts. The manual annotation was performed using a commercially available software tool (EnSite Verismo Segmentation Tool v.2.0.1; Abbott/St Jude Medical, Inc., St. Paul, MN, USA) to manually segment a shell containing the LA body with 4 PVs and the LAA. This whole shell was further parsed (“refined”) into its 6 substructures using 3D Slicer [23], manually. The parsing performance of the DOKEN algorithm was measured by centroid–boundary distances against manual annotations. The CT segmentation performance of the DNN model was measured by Dice scores, average surface distance, and centroid–boundary distances, also against manual annotations.

### 2.5. Performance Evaluation

A newly designed metric, the centroid–boundary distance, was used along with two standard metrics for segmentation tasks [6,7,8,24,25,26,27]—Dice similarity coefficient and average surface distance—to evaluate the model’s accuracy in capturing the 2D LA-PV/LAA boundaries, the global 3D structures, and the local 3D shapes and contours, respectively. Mathematically, the centroid–boundary distance is calculated as the average of all the distances from the centroid of the heart to points on the LA-PV/LAA boundary. The Dice similarity score measures spatial overlap between the model prediction and the ground truth, while 0 indicates no overlap and 1 indicates complete overlap, which can be mathematically expressed as

$$Dice\;Similarity\;Score=\frac{2\times True\;Positive}{2\times True\;Positive+False\;Positive+False\;Negative\;}.$$

The average surface distance is calculated as the average of all the distances from points on the boundary from model prediction to the ground-truth boundary. The success rate of the *DOKEN* algorithm was also calculated, where success was defined as an intersect over union (IoU) between the algorithm prediction and expert manual annotation larger than 0.5. This metric has been widely used for detection tasks [28].

### 2.6. Statistical Analysis

Continuous data are expressed by mean ± SD and categorical data by percentages. The distance and Dice scores were summarized as medians and interquartile range (IQR). Pearson correlation’s test was used to assess the similarity of LA volumes and the LA sphericity index estimated from model prediction and ground truth. The Student’s *t*-test, Chi-square test, or McNemar’s test was applied as appropriate. *p* < 0.05 was considered significant.

## 3. Results

### 3.1. DOKEN Algorithm Can Robustly Parse Cardiac Geometry

**Figure 5:**
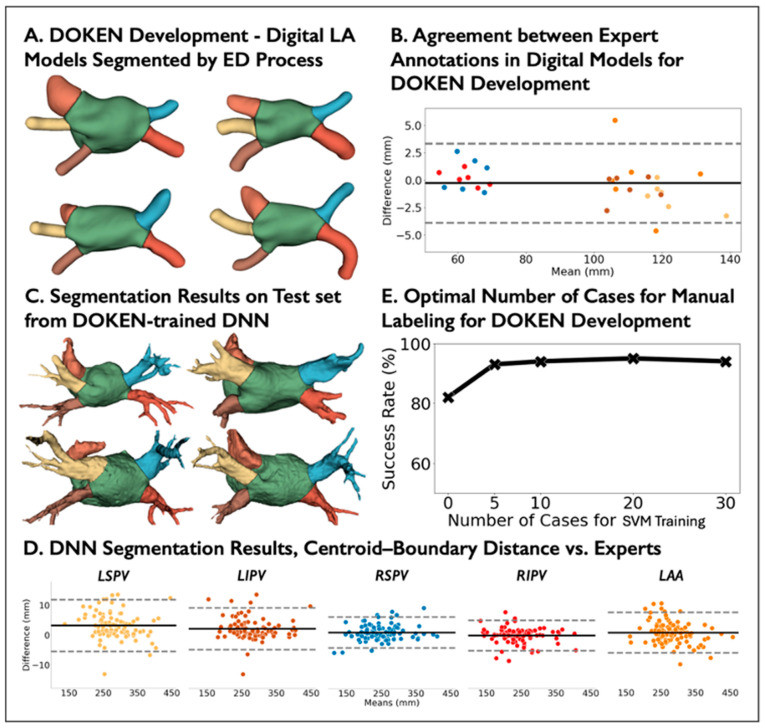
Evaluation of the DOKEN algorithm and the DNN performance for CT image segmentation. (**A**) Examples of digital LA models segmented by the DOKEN algorithm. (**B**) Bland–Altman plot of centroid–boundary distance of *N* = 6 digital LA models segmented by DOKEN compared to experts. (**C**) Examples of patient LA models segmented by the DOKEN algorithm. (**D**) Bland–Altman plot of centroid–boundary distance of *N* = 100 patient LA models in the test set segmented by DOKEN compared to experts. (**E**) Success rate of DOKEN algorithm with different seed cases for SVM training. Refer to panel D for color codes for the plot in panel B.

In digital hearts, the novel DOKEN approach separated the PVs and LAA from the left atrial bodies (Figure 5A) with a mean difference for the centroid–boundary distances of −0.27 mm (95% CI: −3.87 to 3.33; r = 0.99; *p* < 0.0001; Figure 5B). Randomly, five shells of seed data was selected from the *N* = 5 digital atria for tuning, with LA sizes from 71 to 140 mL that cover a broad range of patients [29].

In the test set (*N* = 100), the performance of the tuned DOKEN algorithm was compared to expert annotations. Figure 5C presents example results on the test set. The DOKEN method produced a mean difference and limits of agreement for the centroid–boundary distance of 1.46 mm (95% CI: −5.58 to 8.49; r = 0.99; *p* < 0.0001; Figure 5D). The success rate of the algorithm’s parsing when adding more seed data for tuning was assessed. As shown in Figure 5E, the success rate increased from 67% (no tuning) to 94% by tuning with *N* = 5 shells of seed data (*p =* 0.034; McNemar’s test) and then showed only modest changes (consistency) when tuning in 10–30 shells (92–94%), justifying the selection of seed number.

### 3.2. DNN Trained by DOKEN-Labeled Samples Can Accurately Segment CT

**Figure 6:**
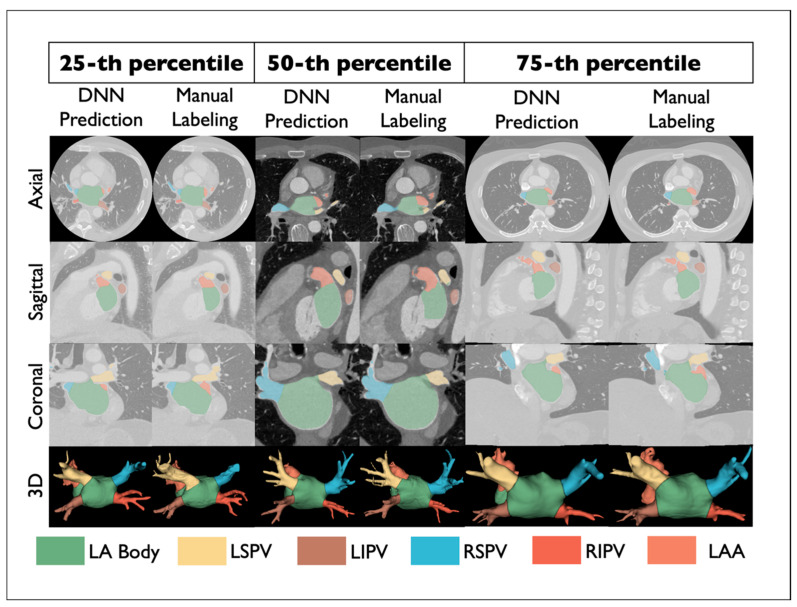
Example results showing DNN segmentation and manual annotation by experts.

**Figure 7:**
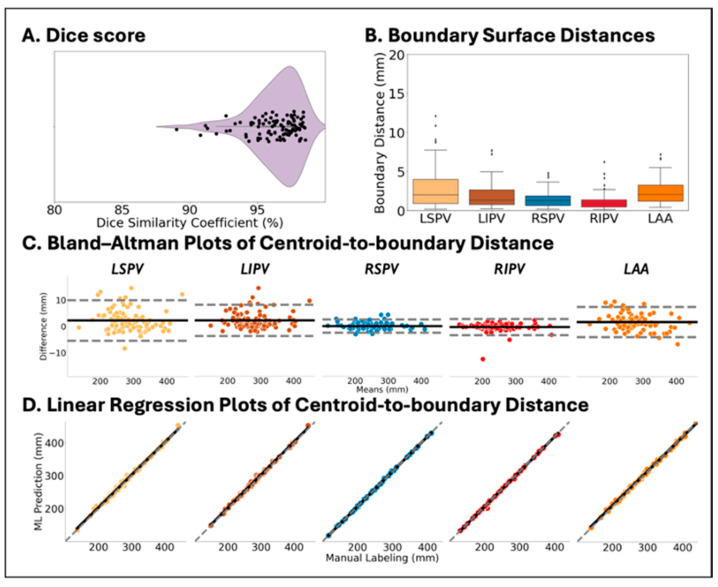
Accuracy of CT image segmentation between DNN prediction and expert labeling in the test set (*N* = 100). (**A**) Violin plot of mean Dice score. (**B**) Box plot of the surface distance of boundaries of 4 PVs and LAA. (**C**,**D**) Bland–Altman and linear regression plots of centroid–boundary distance of 4 PVs and LAA.

Figure 6 shows comparisons between DNN prediction (left) and manually labeled (right) atria from select samples representing the 25th, 50th, and 75th percentile accuracy in the hold-out set (*N* = 100). The Dice score was 96.7% (IQR: 95.3% to 97.7%, Figure 7A), with a median error in surface distance of boundaries of 1.51 mm (IQR: 0.72 to 3.12, Figure 7B) and a mean centroid–boundary distance of 1.16 mm (95% CI: −4.57 to 6.89, Figure 7C), again similar to expert results (r = 0.99; *p* < 0.001, Figure 7D).

Thus, this approach enabled a >10-fold reduction in the relative ratio of training to test cases, inverting the ratio of training:test cases to less than 1:5 from a typical ratio of >3:1.

### 3.3. Analysis of Anatomical Variants

As previously noted, real CT data have more heterogeneity than digital models, such as variation in patient anatomies. Some anatomies could, in fact, be outliers, i.e., their shape does not follow the typical configuration identified in clinical studies. As no pre-screening was performed to eliminate such anatomy variants, it was analyzed if and how variation in anatomies would affect the method’s performance.

**Figure 8:**
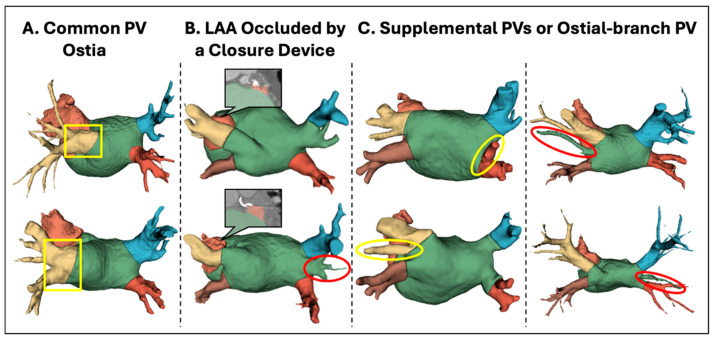
Robust segmentation performance of anatomical variants by the DOKEN algorithm. Three main variants were identified: (**A**) common left PV ostia (*N* = 12), (**B**) LAA occlusion by a closure device (*N* = 3), and (**C**) supplemental PVs or ostial-branch PV (*N* = 25). DOKEN successfully parsed 28/34 of the identified variants (boxed/circled in yellow). However, it missed some extra PVs or branches in the remaining cases (circled in red).

Overall, 100% cases with four PV ostia (the most common anatomic configuration, representing 66 cases) were parsed with boundary distances of 1.26 mm (95% CI: −5.15 to 7.68; r = 0.99; *p* < 0.0001). Three main outlier variants were identified (Figure 8): (1) common left PV ostia (*N* = 12), which was successfully parsed despite a lack of specific training on such cases; (2) LAA occlusion by a closure device (*N* = 3), where residual LAA stumps proximal to the occlusion device were correctly identified despite a lack of specific training in such cases; and (3) supplemental PVs or ostial-branch PV, where the DOKEN algorithm was able to segment 19/25 cases.

In summary, 28/34 of identified variants were successfully parsed with anatomic agreement within 1.95 mm (95% CI: −6.34 to 10.25), which again was in line with expert annotations (r = 0.99; *p* < 0.0001), despite lack of specific training for variants. In the remaining six cases, errors arose mostly from missing PVs or branches relative to the four-PV digital model, which could be addressed by geometric models that adapt to a range of PVs.

## 4. Discussion

Domain knowledge encoding of atrial geometry was able to accelerate a DNN for the segmentation of CT images and enable its training on very small datasets. In this study, the training-to-testing ratio was <1 training to 5 test, which indicates a far lower need for training than the conventional published ratios of >3:1 for ML [8,24,26]. This approach was then tested in a hold-out test set, in which the model accelerated segmentation while maintaining similar accuracy to experts. This novel approach could broaden the ease of access and accuracy of AF ablation. More broadly, this approach has analogies to natural intelligence, which has the potential to reduce the need for large, annotated datasets to train ML and could be applied for diverse applications in imaging as well as 3D printing. A simple post-processing step involving a 3D smoothing operation such as a Taubin filter [30] could extend the proposed work for 3D printing applications (illustrated in Supplemental Figure S1).

### 4.1. DNN Segmentation of Cardiac CT Images

Cardiac CT is increasing used [24,26,31] to guide ablation for AF and to predict clinical endpoints such as the risk of AF recurrence [32,33]. However, the segmentation of these large 70–200 MB datasets manually by experts takes tens of minutes [6,7,8,24] and 4.4–10 min even with latest commercial software such as the CARTO Segmentation Module version 6 (Biosense Webster, Irvine, CA, USA) [34,35]. The present approach greatly accelerates these reports while retaining high accuracy for routine and variant anatomy while achieving competitive accuracy (93.5–96.7%) with previous work (e.g., 91–97% [25] and 93.4% [24]). This study involved a dataset of *N* = 120 patients at a single center. The future extension of this work should expand the study cohort with data from multiple institutions, and the labeling should be further refined using a fusion of annotations from multiple experts and addressing discrepancies by an adjudication committee. One such example is demonstrated in our previous work [36], where we used an independent external dataset to test the performance of the algorithm.

The approach also circumvents the limitation that most CT studies that segmented the LA often did not specifically segment the PVs and LAA [24,26]. Similarly, software tools such as SimVascular (v.2023, https://github.com/SimVascular, accessed on 14 May 2024) provide automatic segmentation, which uses an ML model (CNN) that was trained using a public dataset MM-WHS [7], which only focuses on labels for the chambers but not specifically for the complex substructures such as narrow veins (PVs) and the anisotropic-shaped LAA, which are critical for AF ablation. The DOKEN algorithm, on the other hand, offers a scalable solution to segment complex structures in large medical databases. Further, the DOKEN algorithm’s goal is focused on segmenting intricate cardiac structures and is not intended to be an alternative for advanced tools like SimVascular, which can perform high-fidelity simulations.

Another limitation is the size of publicly available labeled datasets, which are often small, typically provide test cohorts of <40 cases [6,7,8], and may create overfitted ML models that generalize poorly [37]. The DOKEN algorithm enabled training from smaller datasets, inverting the typical ratio of training:test cases and reducing the relative size of training to test cases by 10-fold. This “inversed training–test ratio” paradigm has recently been applied in domains outside medicine such as for Amazon co-purchasing product predictions [38]. Other cardiac imaging applications include the segmentation of magnetic resonance imaging (MRI) data to boost ML by reducing the need for large training data sets.

### 4.2. Challenges in Machine Learning

LeCun et al. and others have stated that difficulties in obtaining large training datasets are among the greatest challenges to machine learning [39]. Obtaining such data is particularly challenging in medicine [40], healthcare [41], and biosciences [42] due to privacy and regulatory requirements. The mathematical encoding of domain knowledge, which emulates some features of natural intelligence, may be a useful approach to address such limitations.

Domain knowledge can be applied in diverse ways. Databases and anatomic atlases have long been used for image segmentation [43,44] but do not encode knowledge principles in a fashion that could be generalized by learning algorithms. Indeed, Trutti et al. [44] pointed out that atlases may identify only a fraction of important structures (7% of 455 subcortical nuclei in the brain), and it is not clear how such “flat” data could be used to identify variants, as we demonstrated. Encoding anatomical knowledge also de-emphasizes low-level details while maintaining high-level abstract information, which may be central to human cognition [12]. The extent of detail required for mathematically encoding is unclear and should be defined for separate applications. Domain knowledge encoding need not be restricted to anatomy and could be applied to processes such as cellular metabolism and physician diagnostic patterns or reports [15].

Alternative approaches are being studied to circumvent large training datasets. Synthetic data may be generated in large quantities to mitigate a lack of actual training data [45], but while they may appear very realistic, they may lack diversity or even introduce bias due to the overfitting [46]. Data augmentation is a widely used approach to training ML on altered versions of the input data to increase the size of the training set [47] but does not capture variations in larger real data [48].

## 5. Conclusions

The novel domain knowledge-encoding algorithm was able to perform the segmentation of six substructures of the LA, reducing the need for large training data sets. The training set had as few as 20 samples, and the hold-out test set included hundreds of patients. The combination of domain knowledge encoding and machine learning approaches could reduce the dependence of ML on large training datasets and could potentially be applied to AF ablation procedures and extended in the future to other imaging, 3D printing, and data science applications.

## Data Availability

The code and networks with trained weights will be released upon acceptance. The 3D digital heart models are publicly available at https://zenodo.org/record/4309958#%23.YdlOJRPMJqs, accessed on 14 May 2024. The CT dataset used in the study is not currently permitted for public release due to the sensitive nature of patient data.

## Supplementary Materials

The following supporting information can be downloaded at: https://www.mdpi.com/article/10.3390/diagnostics14141538/s1, Figure S1: Demonstration of a potential application of our DOKEN algorithm in 3D printing.
