# Supplementary material for: Novel Domain Knowledge-Encoding Algorithm Enables Label-Efficient Deep Learning for Cardiac CT Segmentation to Guide Atrial Fibrillation Treatment in a Pilot Dataset
Source: Diagnostics (Basel). 2024 Jul 17;14(14):1538. doi: 10.3390/diagnostics14141538 (PMC11276420; doi:10.3390/diagnostics14141538)
Supplement: Supplementary file 1 [file diagnostics-14-01538-s001.zip › diagnostics-3035793-supplementary.pdf]

Supplementary Material: Novel Domain Knowledge-Encoding Algorithm Enables Label-Efficient Deep Learning for Cardiac CT Segmentation to Guide Atrial Fibrillation Treatment in a Pilot Dataset

Supplementary Figure S1: Demonstration of a potential application of our DOKEN algorithm in 3D printing.

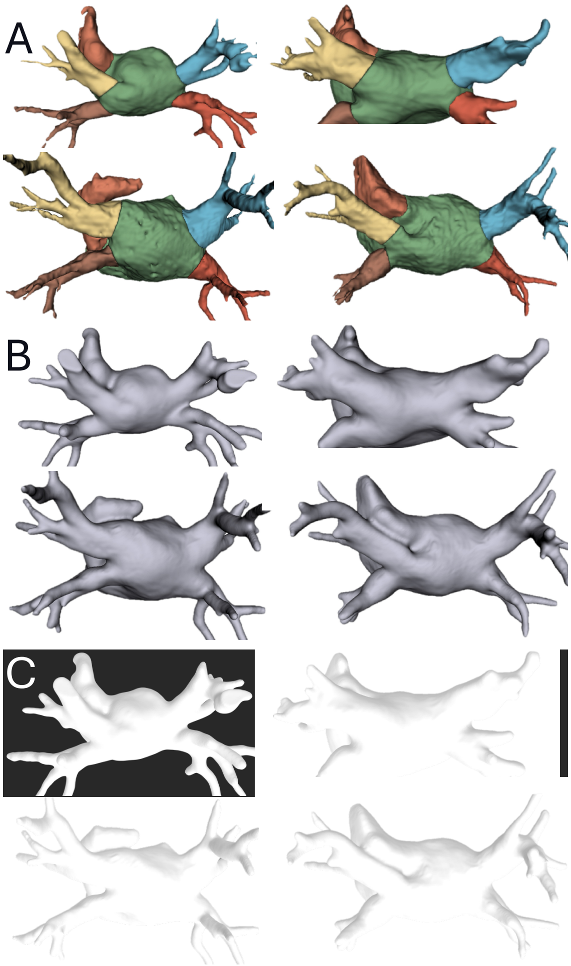

Supplementary Figure S1: Demonstration of a potential application of our DOKEN algorithm in 3D printing. We use the 3D LA models (A) generated by our DOKEN algorithm, which are the same models as demonstrated in Figure 5C, to create smooth STL files. We first use the 3D slicer to generate a uniform-thickness shell defined by the segment boundary using the *Hollow* operation. Subsequently, we apply a *Smoothing* operation to smooth the shell's surface. To streamline the process, we employ the default hyperparameters of the 3D slicer. Finally, we use these smoothed shells (B) to generate the STL files (C), smoothened for 3D printing application.
